# Bacterial community structure and functional contributions to emergence of health or necrotizing enterocolitis in preterm infants

**DOI:** 10.1186/2049-2618-1-20

**Published:** 2013-07-10

**Authors:** Erika C Claud, Kevin P Keegan, Jennifer M Brulc, Lei Lu, Daniela Bartels, Elizabeth Glass, Eugene B Chang, Folker Meyer, Dionysios A Antonopoulos

**Affiliations:** 1 Section of Neonatology, Department of Pediatrics, The University of Chicago, Chicago, IL, USA; 2 Section of Gastroenterology, Department of Medicine, The University of Chicago, Chicago, IL, USA; 3 Institute for Genomics and Systems Biology, Argonne National Laboratory, Argonne, IL, USA; 4 Mathematics and Computer Science Division, Argonne National Laboratory, Argonne, IL, USA

**Keywords:** Necrotizing enterocolitis, Preterm infant microbiome, Metagenomics, Carbohydrate metabolism

## Abstract

**Background:**

Preterm infants represent a unique patient population that is born functionally immature and must accomplish development under the influence of a hospital environment. Neonatal necrotizing enterocolitis (NEC) is an inflammatory intestinal disorder affecting preterm infants. The purpose of this study was to evaluate the progression of intestinal microbiota community development between preterm infants who remained healthy compared to preterm infants who developed NEC.

**Results:**

Weekly fecal samples from ten preterm infants, five with NEC and five matched healthy controls were obtained. Bacterial DNA from individual fecal samples was subjected to sequencing of 16S rRNA-based inventories using the 454 GS-FLX platform. Fecal samples from control infants demonstrated a temporal pattern in their microbiota, which converged toward that of a healthy full term breast-fed infant. Microbiota development in NEC patients diverged from controls beginning three weeks prior to diagnosis. Shotgun metagenomic sequencing was performed to identify functional differences in the respective microbiota of fecal samples from a set of twins in which one twin developed NEC and one did not. The majority of the differentially abundant genes in the NEC patient were associated with carbohydrate metabolism and mapped to members of the family *Enterobacteriaceae*. This may indicate an adaptation of the community to an altered profile of substrate availability for specific members as a first step towards the development of NEC. We propose that the microbial communities as a whole may metabolize milk differently, resulting in differential substrate availability for specific microbial groups. Additional differentially represented gene sets of interest were related to antibiotic resistance and vitamin biosynthesis.

**Conclusions:**

Our results suggest that there is a temporal component to microbiome development in healthy preterm infants. Thus, bacteriotherapy for the treatment or prevention of NEC must consider this temporal component of the microbial community in addition to its taxonomic composition and functional content.

## Background

Under normal conditions, genetics, maternal environment, nutrition, and intrauterine stressors all contribute to fetal development within a protected, sterile, intrauterine environment. However, infants born preterm are unique, as interruption of the *in utero* environment mandates development within the neonatal intensive care unit, under the influence of hospital environment and stressors. This hospital environment results in premature microbial colonization of the infant during critical developmental transitions that normally occur without microbial influence.

Every organ system of the preterm infant is immature and faces its own challenges. For the preterm gut, the challenges include interaction with the intestinal microbiota, processing of food, and the specific disease neonatal necrotizing enterocolitis (NEC). NEC is a devastating inflammatory bowel necrosis that primarily afflicts premature infants after the initiation of enteral feeding. The primary risk factors appear to be prematurity and bacterial colonization [1]. There is a high mortality rate of 20 to 30% and up to 50% in infants who require surgery [2]. Morbidity includes risk of intestinal stricture, short-gut, intestinal failure, and poor neurodevelopmental outcome [3-5]. Thus, there is great interest in investigating means of predicting, preventing, and treating this disease.

Bacteria are believed to be important in the pathogenesis of NEC, however no specific pathogens have been identified. We have previously published 16S rRNA-based sequence inventories of the intestinal microbiota of preterm infants with and without NEC, from fecal samples at time of NEC diagnosis [6]. Clustering based on microbial community composition exhibits distinct separation between NEC patients and corresponding controls. Intestinal bacterial colonization in all preterm infants was notable for low diversity, but samples from patients at the time of NEC diagnosis had even less diversity, marked by an increase in the abundance of Gammaproteobacteria and a decrease in other bacterial species. While this study was the first to demonstrate a clear bacterial pattern associated with NEC, it was limited by its focus on the time of NEC diagnosis and bacterial taxonomy solely inferred from 16S rRNA gene sequences. The present study was designed to examine the development of the intestinal microbial community over time in NEC and control patients. We hypothesized that NEC is not an acute bacterial insult, but rather, infants that eventually develop NEC acquire an altered intestinal microbiota which influences intestinal homeostasis weeks prior to the onset of clinical symptoms.

**Figure 1:**
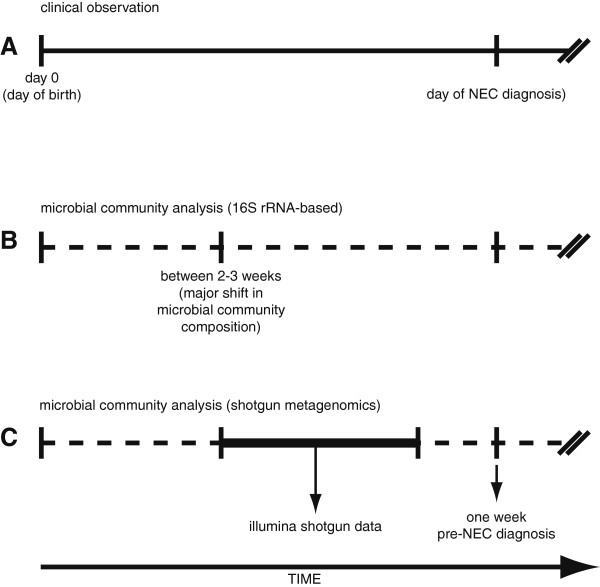
**Timeline of sample collection from NEC and control patients.** Included subjects were born between 24–32 weeks gestation and stool samples were collected weekly from birth to 10 weeks of life. Panel **A** - 16S rRNA-based microbial community analyses were performed on samples from throughout this period and indicated a shift in microbial communities at approximately 2 weeks after birth. Panel **B** - Shotgun DNA sequencing of metagenomes from samples on the day of NEC diagnosis and one week before showed no significant difference. Panel **C** - Subsequent shotgun sequencing of metagenomes focused on the interim period.

If an inappropriate microbial pattern is predictive of NEC, it is important to understand how it differs from that of non-diseased patients and when it develops. Fecal samples were collected prospectively from birth to ten weeks of life from preterm infants < 1500 gm (Figure 1). Sequencing of 16S rRNA-based inventories using the 454 GS-FLX platform identified a progression of microbial community development in preterm infants without NEC with clusters at two weeks, three to five weeks, and ≥ six weeks. Furthermore, samples from patients with NEC diverged from those of control patients three weeks prior to NEC diagnosis. We specifically identified a critical difference in microbial community structure between infants with and without NEC prior to two weeks of life notable for a significant decrease in the phylum Firmicutes in infants who went on to develop NEC compared to controls. Shotgun metagenomics-based analyses were used to examine gene function within these microbial communities. Analysis of fecal samples from a set of twins - one developed NEC, the other did not - demonstrated a clear separation in functional gene sets prior to NEC onset. Gene sets differentially abundant between these two profiles were primarily associated with carbohydrate metabolism; the majority was more abundant in the twin sample prior to NEC onset. Additional differentially represented gene sets of interest were related to antibiotic resistance and vitamin biosynthesis.

## Methods

### Patient characteristics and sample collection

Subjects were recruited from the level III neonatal intensive care unit (NICU) at Comer Children’s Hospital at The University of Chicago. All patients underwent routine NICU care as determined by the managing service. Spontaneously produced fecal samples were collected weekly from birth to 10 weeks of life. As patients did not produce a sample each week, sample collection yielded 56 fecal samples from 10 patients including five infants with NEC and five infants without NEC as control.

Included subjects were born between 24 and 32 weeks gestation. Five males and five females were included. All patients were delivered by cesarean section. One of the infants was exclusively formula-fed, four of the patients were exclusively breast-fed and five received a combination of formula and breast milk. NEC and control patients were matched for gestational age and day of sample collection. Other study patient characteristics were not significantly different between NEC and control groups (Table 1).

**Table 1:** Clinical characteristics of the hospitalized preterm infants in this study

**Characteristics**	**NEC infants (n = 5)**	**Control infants (n = 5)**
Male/female	2/3	3/2
Gestational age (weeks)	24.4 to 32 (mean 26.8)	24.1 to 32 (mean 26.4)
Delivery: c-section/vaginal	5/0	5/0
Feeding: breast milk/formula/combination	2/1/2	2/0/3

The study was approved by the University of Chicago institutional review board for human studies, and informed consent was obtained from patients’ parents. Nurses collected the fecal sample directly from the diaper into the collection tube using the wooden end of a sterile cotton swab. The sample was immediately frozen. All samples were stored at −80°C until processed.

### DNA extraction and metagenome sequencing

Metagenomic DNA was isolated from 200 mg of frozen fecal sample using an amended protocol for the MagNA Pure Compact System (Roche Applied Science). For DNA extraction, 50 mg of the frozen fecal sample was dissolved in 1 ml of extraction buffer (50 mM Tris (pH 7.4), 100 mM EDTA (pH 8.0), 400 mM NaCl, 0.5% SDS) containing 20 μL proteinase K (20 mg/ml). 500 μL of a slurry of 0.1 mm-diameter zirconia/silica beads (BioSpec Products) were added into the extraction tubes and a Mini-Beadbeater-16 (BioSpec Products) was used to lyse the microbial cells for a total of two minutes (two times one minute intervals).

DNA concentration and quality were determined by fluorometry (using the Qubit (Invitrogen)) and verified with agarose gel electrophoresis. Five micrograms of DNA were then used to construct shotgun libraries for sequencing using the GS-FLX LR70 and XLR70 sequencing chemistry (454 Roche Applied Science) as well as for the Illumina GAIIx platform (Illumina Inc). Sequencing was performed at the High-Throughput Genome Analysis Core (HGAC; part of the Institute for Genomics & Systems Biology (IGSB)) at Argonne National Laboratory.

### 16S rRNA-based amplicon library preparation and data analysis

Amplicon libraries targeted the 16S rRNA encoding gene to obtain deep surveys of the microbial communities using the GS-FLX platform and XLR70 sequencing chemistry. PCR primers specific for the V3-V4 region of the 16S rRNA encoding gene (*Escherichia coli* positions 338 to 802) containing 454-specific adapter sequences as well as an 8-base pair barcode were utilized. This barcode-based primer approach allowed sequencing of multiple samples in a single 454 sequencing run without the need for physical partitioning.

Processing of the 16S rRNA-derived sequence inventories was performed using the QIIME toolkit (QIIME 1.5.0) [7]. Briefly, OTUs were selected at 97% sequence identity using uclust and a representative sequence was then chosen for each OTU based on the most abundant sequence in that OTU. Representative sequences were then aligned using PyNAST, and a taxonomic classification was assigned to representative sequences using the RDP Classifier. These PyNAST-aligned sequences were also used to build a phylogenetic tree with FastTree, and unweighted UniFrac distances were then calculated between all samples.

### Shotgun metagenomic analysis and statistical analyses

The metagenome data were analyzed using the MG-RAST system [8], which provides the ability to annotate with respect to a number of existing databases through use of the M5nr [9] - we selected subsystems-based annotations [10]. Initially, sequence fragments undergo a quality control and de-replication step (exact duplicates being a sequencing artifact). Next, the sequences are screened against multiple functional (protein) and taxonomic (16S rRNA-based) databases via BLAT-based comparison to the MG-RAST M5nr [9] for potential protein encoding genes (PEGs) and other features (for example, rRNA-based ones). A phylogenomic-based reconstruction (taxonomic inference from the nearest matching protein) of the sample was then computed by using the taxonomic information associated with the match in the M5nr [9]. The data are publicly available via the MG-RAST server based at Argonne National Laboratory (http://metagenomics.anl.gov/), including instant availability of the sequence data, bioinformatic analyses and tools, plus the support for metadata features encoded using minimum information about a (meta)genome sequence (MIGS/MIMS) [11].

Subsystems count data for each sample metagenome were log transformed and centered to facilitate parametric tests. Principal component analysis (using the variance-covariance matrix, since all of the relative sequence abundance based on the proportion of annotated subsystem was similar for all sampling units) was used to compare condensed subsystems data among samples. These analyses were performed in R using the packages Stats and pcaMethods (http://r-project.org/; http://cran.r-project.org/). Results were considered significant at α = 0.05 with Bonferroni correction; a stringent adjustment was intentionally chosen to demonstrate the robustness of trends.

## Results

### 16S rRNA-based microbial community analysis

As an understanding of the temporal development of the microbial community was a critical aspect of the present study, fecal samples were collected prospectively on a weekly basis and analyzed by 16S rRNA-based sequencing. Patient characteristics (listed in Table 1) were not significantly different between NEC and control groups. Amplicon libraries were PCR amplified, targeting the V3-V4 region of the 16S rRNA gene using 338F and 802R primer sets. Libraries were then sequenced using 454 XLR70 sequencing chemistry and analyzed with the QIIME software package [7]. On average, approximately 5,700 sequences were generated per library (detailed sequence outputs for libraries are listed in Additional file 1: Table S1). Following binning of the sequences into operational taxonomic units (OTUs) based on 97% sequence identity, comparisons were made using principal coordinates analysis (PCoA) based on unweighted UniFrac distances. The dominant phyla in the samples were Proteobacteria and Firmicutes and to a lesser degree Actinobacteria.

**Figure 2:**
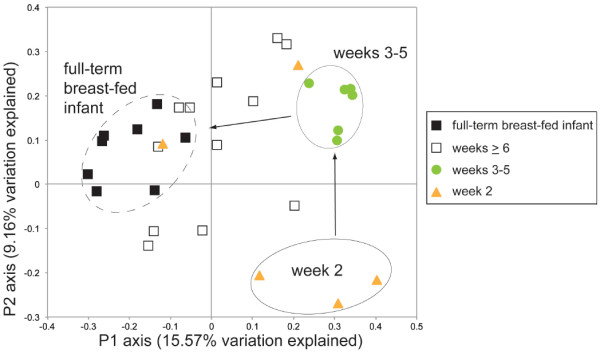
**16S rRNA**-**based analysis of samples demonstrates a temporal component to healthy development in preterm infants.** Samples from a full term breast-fed infant (solid black squares) cluster distinctly from early stages of preterm infant development (up through five weeks of life) along the first principal axis. Additional clusters are detected along the second principal axis (within two weeks of life (orange triangles), between three to five weeks (green circles) along the second axis) denoting a temporal progression (arrows) of the microbial communities towards a stable healthy state represented by the full term breast-fed infant.

Development of the microbial communities in the control cohort over time was examined and compared to results from a similar set of prospectively collected fecal samples from a healthy full term breast-fed infant cared for at home (Figure 2). The samples corresponding to microbial communities from the full term breast-fed infant (black filled-in squares) clustered tightly and separated distinctly on the first principal axis (P1) from preterm infant communities sampled at early time points (week two and weeks three to five clusters). A developmental shift in the preterm infant microbial community was detected among earlier time points on the second principal axis (P2) exhibited by the tight clustering of the microbial communities from weeks three to five (green circles) relative to the week two derived communities (orange triangles). The preterm infant communities from later time points (weeks ≥ 6; open squares) exhibited a range of values crossing the first principal axis intermediate between the early preterm infant and full term infant samples, suggesting a developmental trajectory (arrows) leading from an immature to a mature and healthy full term infant microbial community state.

**Figure 3:**
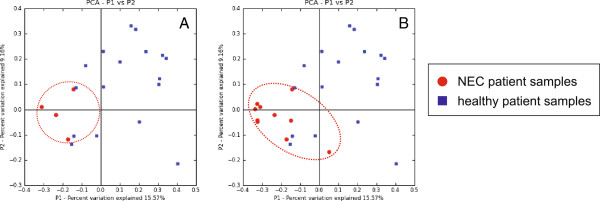
**16S rRNA**-**based analysis demonstrates deviation from a healthy state by NEC patients prior to diagnosis. (A)** PCoA of control patients after two weeks of life (blue), and NEC patients between three weeks prior to NEC diagnosis up to date of diagnosis demonstrate a separation of the NEC patients. **(B)** PCoA of control patients after two weeks of life (blue), and NEC patients (red) between three weeks prior to NEC diagnosis and onwards past diagnosis demonstrate increasing clustering and deviation of the NEC patients.

When samples obtained from NEC patients were compared with the controls, microbial communities from patients that subsequently developed NEC (in red; Figure 3A) were distinct from control samples (blue; Figure 3A) three weeks prior to diagnosis. Continued analysis of the microbial communities from these same patients - addition of time points following NEC diagnosis - reinforced the observed divergence of the NEC microbial community when compared to the controls (Figure 3B), particularly on the first principal axis (P1; ANOSIM *R* = 0.6388, *P* = 0.001). In both patient data sets, the predominant phylum represented was Proteobacteria with members of the Firmicutes in the minority. However, prior to two weeks old, the control infants had a much higher percentage of Firmicutes compared to those infants who went on to develop NEC (49.9% compared to 25.0%, respectively).

### Shotgun sequencing of microbial communities

As it appeared that a skewing in bacterial community composition occurred in patients prior to NEC diagnosis, we asked if a corresponding difference in the functional gene content of the community also occurred. In order to more closely investigate functional consequences of the altered microbial communities observed prior to the onset of NEC, shotgun metagenomic sequencing was conducted on samples collected between the time of the first bacterial community shift (highlighted by the arrow between two to three weeks of life as described in Figure 2) up to NEC diagnosis (Figure 1). 454-based pyrosequencing was initially applied to fecal samples from a small cohort of patients to generate shotgun metagenomes. Sequence data were annotated using the MG-RAST (MetaGenome Rapid Annotation using Subsystems Technology) analysis platform (http://metagenomics.anl.gov; Meyer *et al*., 2008). Samples collected one week prior to NEC diagnosis were indistinguishable from those collected on the day of NEC diagnosis (data not shown). Guided by the 16S rRNA-based inventories (Figures 2 and 3), and to see if the development of a distinct NEC functional profile could be observed before diagnosis, subsequent shotgun sequencing focused on earlier time points (three weeks prior to NEC diagnosis).

Samples from twin patients (one healthy and one diagnosed with NEC) at two time points prior to NEC diagnosis were subjected to ultra-deep sequencing using the Illumina GAIIx platform. Early samples from each twin (three weeks prior to NEC diagnosis) were compared to a second time point; for the twin that developed NEC this was 10 days prior to NEC diagnosis (noted as ‘pre-NEC’ sample). Paired-end libraries were sequenced yielding an average of 4.08 ± 0.46 Gb per sample. Following standard sequence preprocessing in the MG-RAST pipeline for sequence length and quality, including removal of artifactual replicate reads, just over 14 million sequences (14.06 × 10^6^ ± 0.79 × 10^6^ sequences) were available for annotation per sample (Additional file 1: Table S2). Functional annotation abundance profiles were then subjected to statistical analyses.

**Figure 4:**
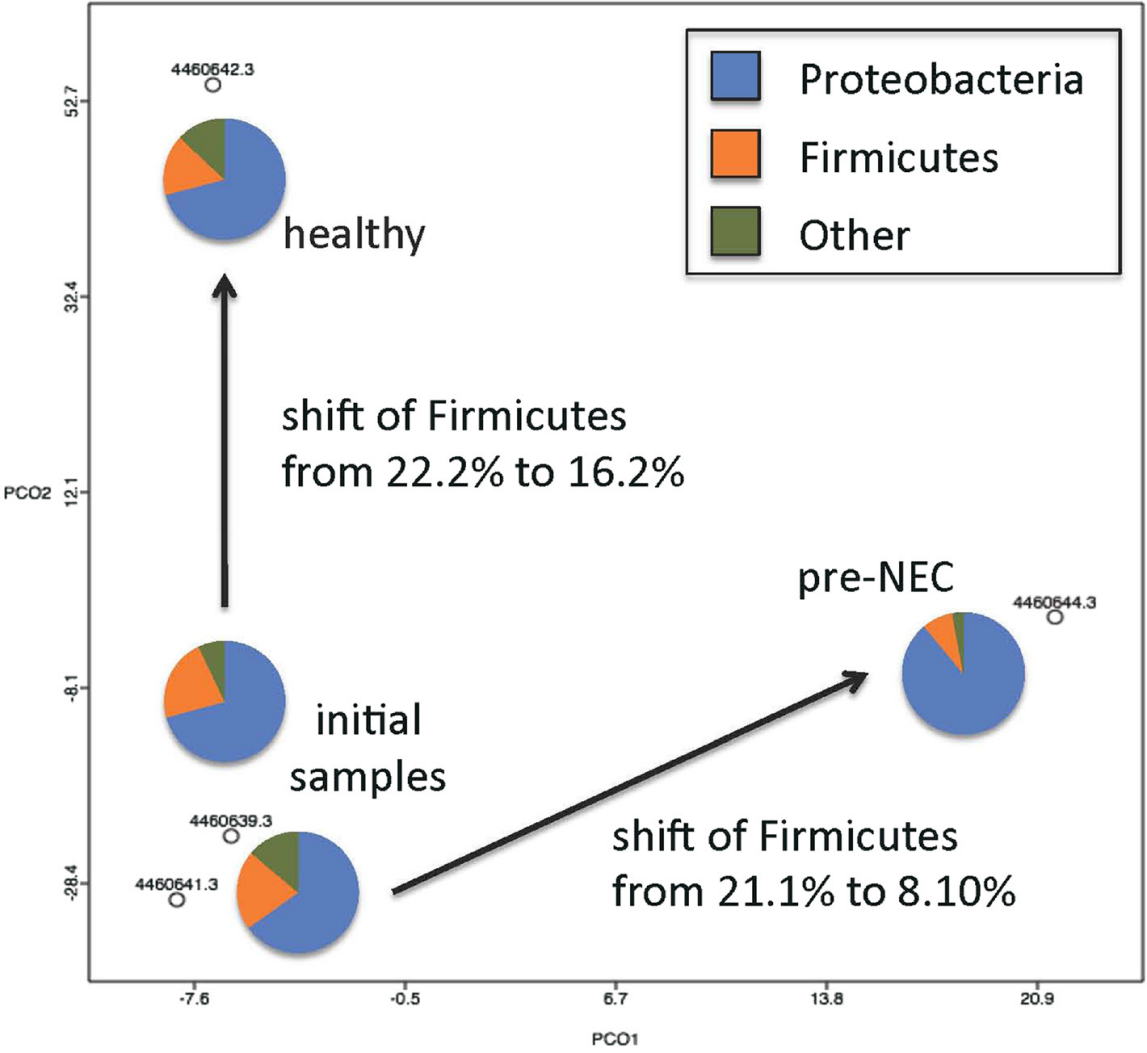
**Analysis of microbial communities by shotgun metagenomics between two weeks of life and NEC diagnosis demonstrate functional distinction.** Shotgun metagenomes generated from twin patients at times prior to NEC diagnosis (only one of the twins went on to be diagnosed with NEC; labeled ‘pre-NEC’). An expansion of the Proteobacteria is noted in the patient that went on to develop NEC.

Principal component analysis (PCA) was used to compare the four datasets based on the subsystems ontology for gene annotation [10]. The first time points from both twins clustered together in the PCA plot (Figure 4). The second time points diverged from the initial set; however, while the second time point from the healthy twin remained unchanged on the same first principal axis, the pre-NEC sample from the twin ultimately diagnosed with NEC diverged significantly along this same axis, indicating a distinct metagenomic profile from the other three samples (arrows Figure 4). In all four datasets, members of the *Enterobacteriaceae* were the dominant family (Proteobacteria phylum), however for the pre-NEC dataset there was a noticeable increase in the dominance of this family. This was accompanied by a marked decrease of members of the Firmicutes, specifically the lactate-fermenting genus *Veillonella*, a member of the *Veillonellaceae* family. Additionally, presumed sulfate-reducing bacteria (SRBs; members of the Firmicutes family *Peptococcaceae* including *Desulfitobacterium* and *Desulfotomaculum*) were detected at low levels (0.26% for the healthy twin at the first time point, and 0.27% at the second; 0.28% for the NEC twin at the first time point but 0.058% at the pre-NEC time point).

**Figure 5:**
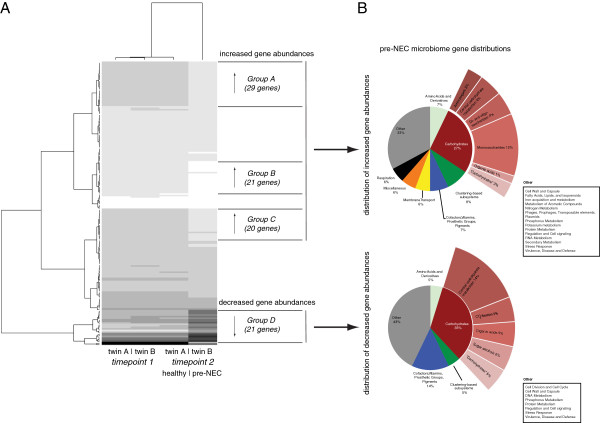
**Differences in gene abundances between pre**-**NEC sample versus other samples.** Heat map **(A)** and pie charts **(B)** displaying significant differences in gene abundances between later pre-NEC time point of NEC diagnosed patient versus other samples.

An array of correlated subsystems appears to distinguish the sample shortly before NEC diagnosis and the other three samples analyzed, based on ANOVA of the respective annotated gene abundance profiles from the pre-NEC sample compared to the other three samples (Figure 5A). Although representative functional roles were identified from almost all of the major level 1 subsystems classes, over 60% of the functional roles identified by ANOVA (*P*-value < 0.005) between the pre-NEC sample and the other three samples fell into the following major subsystems: carbohydrates (20.0%); clustering-based subsystems (10.3%); cofactors, vitamins, prosthetic groups, pigments (7.03%); membrane transport (6.49%); miscellaneous (6.49%); amino acids and derivatives (5.41%); and respiration (5.41%). We further characterized the abundance profiles of the annotated genes by Ward’s clustering, revealing three large clusters of genes (gene sets A, B, and C) that were significantly (*P*-value < 0.005) more abundant in the pre-NEC sample (as noted in the heat map; Figure 5A) and one cluster that was significantly less abundant relative to the other three samples (ratio < 1.0; gene set D).

#### Carbohydrate utilization differences

Over half of the carbohydrates subsystems identified as significantly different in their abundance between the second time point from the NEC diagnosed twin (10 days prior to NEC diagnosis; ‘pre-NEC’ sample) and the other three samples were increased. These subsystems were related to monosaccharides (comprising 5.41% of the total functional roles identified using ANOVA with a *P*-value < 0.005), central carbohydrate metabolism (4.32%), and di- and oligosaccharides (3.24%; Figure 5B). The majority of the carbohydrate-associated genes were found in gene sets A and D with significant representation encompassing almost half of gene set A (14 out of 29) and one third of gene set D (7 out of 21). Few of the genes encoding central carbohydrate metabolism (pyruvate carboxyl transferase [EC 6.4.1.1] and two forms of glyoxylate reductase [EC 1.1.1.26; EC 1.1.1.79]) were significantly decreased in this same sample.

#### Annotated genes encoding for cofactors, vitamins, prosthetic groups, and pigments

Of the cofactors, vitamins, prosthetic groups, and pigment subsystems represented within the Group A of higher abundance genes detected in the pre-NEC sample, two sets were associated with tetrapyrroles (specifically heme and siroheme biosynthesis; precorrin-2 oxidase [EC 1.3.1.76] and sirohydrochlorin ferrochelatase [EC 4.99.1.4]) while the third was associated with folate and pterines (YgfZ; S-(hydroxymethyl)glutathione dehydrogenase [EC 1.1.1.284]). Within the Group C set of higher abundance genes only those identified as pyridoxine related were detected (pyridoxine (Vitamin B6) biosynthesis; 4-hydroxythreonine-4-phosphate dehydrogenase [EC 1.1.1.262]).

Within Group D, representing the lower abundance genes detected in the pre-NEC sample, three sets of annotated genes were detected associated with tetrapyrroles. These included those for cobalamin synthesis (cobalt-precorrin-8× methylmutase [EC 5.4.1.2]), coenzyme B12 biosynthesis (cobalt-precorrin-2 C20-methyltransferase [EC 2.1.1.130]), and heme and siroheme biosynthesis (hemoprotein HemQ, an essential component of the heme biosynthetic pathway in Firmicutes and Actinobacteria).

## Discussion and conclusions

The development of bacterial patterns in preterm infants has not been previously reported. Interactions between the preterm infant and its enteric bacteria are unique. The preterm infant is quite different from the full term infant as the preterm infant possesses an immature gut that must complete development in the extrauterine environment. Additionally, the preterm infant acquires its microbiota within the confines of the neonatal intensive care unit where colonization is significantly influenced by iatrogenic manipulations including: a hospital environment; frequent use of broad spectrum antibiotics, opioids, and histamine-2 receptor antagonists (H2 blockers); and instrumentation with endotracheal tubes, feeding tubes, and suctioning tubes. It is possible that these nosocomial interactions alter the preterm infant’s endogenous microbiota, thereby influencing development of the immature preterm gut and its susceptibility to NEC.

Our data suggest that development of the microbiota in preterm infants differs markedly between patients that do or do not ultimately develop neonatal necrotizing enterocolitis. Unifrac-based PCoA of the 16S rRNA-based 454 sequence data reveals clear temporal separation between the early samples (< 6 weeks of life) in the control preterm infants and those from the healthy full term breast-fed infant along the first principal component axis. These observations suggest a developmental component to both the microbial community as well as host physiology. Interestingly, samples from the controls at ≥ 6 weeks of life, cross the first principal component axis and appear to converge toward the microbiota pattern of the healthy full term infant. If the microbiota of the healthy full term infant represents a necessary complement of microbiota for gut function, clustering of later time point preterm microbiota with this community may represent development towards a native, healthy microbiota.

Samples from preterm control infants of all gestational ages followed the above temporal progression, suggesting that microbiota development occurs over a requisite period of time rather than correlating with a specific gestational age. This microbial community difference may represent the result of clear differences in the environment of the neonatal intensive care unit as outlined above, or an immaturity in the intestinal epithelium. Previous studies have noted that immature intestinal epithelium has decreased intestinal motility, altered surface glycoconjugate glycoslation patterns and altered intestinal mucus density and carbohydrate content, all of which may alter bacterial binding [12,13].

In contrast, comparison of NEC and control patients demonstrates a distinct trajectory of community development in patients that develop NEC. The ultimate trajectory of microbiota in these infants does not overlap with that of control infants, suggesting that at crucial times a healthy trajectory can be misdirected towards deviant states (for example, NEC). The deviation from control appears to occur two to three weeks prior to NEC. Previous work by others [14] had noted a similar deviation from healthy infants but only at one week prior to the onset of NEC. Earlier detection, as demonstrated here, would provide an extended timeframe for intervention, but is also indicative of a more prolonged microbial community deviation prior to pathology emergence. Additionally, the earlier detection of this trajectory also led us to sequence metagenomes from earlier time points (three weeks prior to NEC diagnosis) and allowed the identification of functional gene sets that may contribute to the onset of NEC. The initial time point from the set of twins we sampled coincided with the two to three week transition period that we identified in the original control cohort (Figure 2). Although the 16S-based data indicate a shift between controls and those that go on to develop NEC, the first time point comparison between twins, based on ordination of the functional genes detected in the metagenomes does not reflect a major difference. This may be explained by organisms that differ at taxonomic resolutions finer than those reported here, occupying a similar ecological niche between patients at this first time point. In other words, there is a functional redundancy between the community memberships wherein different organisms can perform the same community function. Our present efforts are a coarse level description of functional differences between samples that will provide a candidate set of functions to investigate at a finer scale resolution in subsequent work.

This pattern of deviation prior to onset of NEC is reiterated in our shotgun metagenomic data from twins (Figure 4). NEC may result from the presence of a pathogenic pattern, or alternately, the absence of a beneficial one. Reads from each sample were recruited to available sequenced genomes including candidate pathogens. In all four datasets, the most reads recruited (average of 15.07 ± 2.23% post QC reads) were to the genome from *Escherichia coli* strain K-12 (Additional file 1: Table S3). Recruited reads to known pathogens (*Shigella* sp. D9, *Citrobacter koseri*, *Klebsiella pneumoniae* subsp. *pneumonia*, *Yersinia pestis*, and *Cronobacter sakazakii*) were substantially lower but consistent across samples (Additional file 1: Table S3) from both patients indicating a diminished role in development of NEC.

To examine differences in the potential function of the microbiota in NEC versus control patients, metagenomic analysis of samples prior to the incidence of NEC was conducted on a pair of twins, one of which went on to develop NEC. Evaluation of this set of twins enabled us to control for environment (neonatal intensive care unit, and diet) and host factors (genetics), allowing us to observe the development of the microbial community over time, with minimal bias introduced by environment or background genetics.

Functions that were statistically significant were dominated by carbohydrate associated genes that mapped to the *Enterobacteriaceae* (Figure 5). These may represent differences in host metabolism, altering substrate delivery to downstream microbes, or may represent differences in inherent bacterial metabolic pathways that may influence production of metabolites such as short chain fatty acids that affect gut function. Most of the carbohydrate genes identified as significantly distinct between the pre-NEC patient sample and the other three samples were enriched within the pre-NEC dataset.

Only pyruvate carboxyl transferase (EC 6.4.1.1), and two forms of glyoxylate reductase (EC 1.1.1.26; EC 1.1.1.79) were found in lower abundance in the pre-NEC sample. Both of these gene sets are part of the anaplerotic sequences (replenishing reactions) for the tricarboxylic acid cycle to provide precursors for biosynthesis. Pyruvate carboxyl transferase catalyzes the conversion of pyruvate to oxaloacetate while glyoxylate reductase is an enzyme in the oxalate synthesis pathway responsible for converting glyoxylate to glycolate [15]. Alterations in these gene sets may be markers of the health of the microbial community. As part of the anaplerotic reactions, both genes are upregulated when an organism is subsisting on substrates other than glucose.

Microbial communities may metabolize milk through alternative paths, resulting in differential substrate availability for specific microbial groups. The observation of the significantly lower representation of these particular gene sets in the pre-NEC dataset, in combination with an overall enrichment of genes involved in the utilization of carbohydrates, may reflect a change in substrate availability for specific community members presaging the onset of NEC. The pre-NEC community exhibits an expansion and enrichment of these gene sets that were not present earlier, stabilizing its ability to adapt to changes in substrate availability. Furthermore, the distinct profile of functional genes that are enriched in the pre-NEC sample (relative to the three healthy controls) reflects a narrowing of the overall microbial community diversity.

Alterations in these gene sets may also result in bacterial products that have significant effects on the host. For example, a deficiency in pyruvate carboxylase results in conversion of excess pyruvate to lactate. In the infant gut, lactate is used by the intestinal mucosa for gluconeongenesis to supply the small intestinal muscle with glucose [16]. Additionally, a deficiency in glyoxylate reductase may result in increased levels of glyoxylate which has been shown to inhibit the interaction of the Vitamin D receptor (VDR) with vitamin D response elements (VDREs) [17]. Vitamin D has been shown to be involved in the pathogenesis of inflammatory bowel disease and to have immunomodulatory effects [18].

Genes annotated as cofactor, vitamin, prosthetic group, and pigment subsystems were found to be in higher abundance in the pre-NEC sample. These were primarily associated with tetrapyrroles, specifically, heme and siroheme biosynthesis (precorrin-2 oxidase [EC 1.3.1.76] and sirohydrochlorin ferrochelatase [EC 4.99.1.4]). Precorrin-2 oxidase is a multifunctional enzyme that catalyzes the SAM-dependent methylation of uroporphyrinogen III to form precorrin-2 and trimethylpyrrocorphin 2 and also the subsequent conversion of precorrin-2 into siroheme. Siroheme plays a major role in the sulfur assimilation pathway: converting sulfite to a biologically useful sulfide. The etiology of ulcerative colitis is uncertain but may relate to environmental factors in genetically predisposed individuals. Sulfate-reducing bacteria (SRB) have been implicated through the harmful effects of hydrogen sulfide, a by-product of their respiration, as it is freely permeable to cell membranes and inhibits butyrate oxidation by the host. However, inspection of the taxonomic assignments of the annotated genes (via phylogenomic inference) across all four samples revealed a lower relative abundance of known SRBs when compared to the other three samples, indicating that a sulfide-mediated phenomenon may not be a key requirement for the onset of NEC.

Within the virulence, disease and defense subsystems, genes encoding for resistance to antibiotics and toxic compounds were detected within both the Group B and Group D gene sets. Those detected in the Group B set (over-represented in the pre-NEC sample) are part of the *mdtABCD* multidrug resistance gene cluster (encoding for the multidrug transporter MdtC), which confer resistance to deoxycholate and novobiocin. In the Group D set, which contains those genes that are under-represented in the pre-NEC sample, the resistance genes detected are related to the BlaR1 protein family (specifically peptidase M48, Ste24p precursor). This group of genes encodes a family of membrane-spanning proteins with a penicillin sensor and a signal transducer domain that provides the starting point for this resistance mechanism. While utilization of neither antibiotic is noted in the clinical records for the patient, deoxycholate is a bile salt and may be indicative of a community response to changes in the intestinal contents.

It has been suggested that premature infants would benefit from optimized intestinal bacterial colonization; however ‘optimal’ colonization has not been defined. Our data suggest that the colonization pattern of the healthy full term breast-fed infant may represent this optimal colonization for the preterm infant as well. While several studies have evaluated the progression of colonization patterns in healthy full term infants, and have demonstrated that there is high variability between colonization patterns of individual infants at early ages, some patterns have emerged [19]. Previous sequencing based studies have demonstrated that immediately after vaginal delivery, infants have colonization patterns reflective of the maternal vaginal compartment including *Lactobacillus*, *Prevotella*, or *Sneathia* species [20]. Culture-based studies have then shown that these infants have earlier colonization with *Bifidobacterium* and *Lactobacillus*. Breast-feeding is associated with colonization by *Bifidobacterium, Lactobacillus* and *Streptococcus*[21-25]. Several clinical studies have shown a beneficial effect of probiotics on the incidence of NEC [26-28]. In these studies, probiotic administration was begun early with primarily Bifidobacteria and Lactobacillus species that may have altered the overall community toward the healthy breast-fed infant community profile.

Our data suggest that microbial community developmental patterns are important for health. It is possible that early patterns induce key developmental pathways critical for protection of the preterm infant. Studies have shown that bacteria can influence maturation in many ways - prevention of apoptosis, improvement of intestinal barrier function, establishment of intestinal mucus, maturation of glycoconjugate patterns, improvement in immune response, and maturation of intestinal vasculature patterns. All of these may be important for health and protection against NEC. Further testing beyond the scope of this study is necessary to confirm the mechanistic implications of the microbiota differences found for NEC pathogenesis. The host response to these microbiota differences, also warrants further study. However, since bacterial patterns differ between control and NEC patients, our data suggests that bacterial therapy may be an option for NEC prevention. Our studies suggest that a shift toward the microbial patterns seen in control infants may be protective. Additionally, our data suggest that identifying a shift in microbial community three weeks prior to NEC could be used to predict patients at risk for NEC. As such, our data emphasizes the importance of the temporal nature of the microbial community development in preterm infants. Identifying the critical time point, key microbial components, and functions will be necessary for effective bacterial therapeutics to prevent NEC. In the neonatal population, microbial patterns are not yet established; thus, in this patient group one has the unique opportunity to initiate and influence colonization. Our data demonstrate a difference in microbial community structure by both taxonomy and function several weeks prior to NEC. This suggests a window of opportunity in which interventions to alter community structure could influence clinical outcome.

## Availability of supporting data

Both 16S rRNA-derived data and shotgun metagenomic data are available through a project specific page via the MG-RAST server (http://metagenomics.anl.gov).

## Abbreviations

16S rRNA: 16S ribosomal RNA; BLAT: BLAST (basic local alignment search tool)-like alignment tool; M5nr: M5 (metagenomics metadata, meta-analysis, models and meta-infrastructure) non-redundant protein database; MIG/MIM: Minimum information about a genome/metagenome sequence; MG-RAST: Metagenomics RAST (rapid annotation using subsystems Technology); NEC: Necrotizing enterocolitis; NICU: Neonatal intensive care unit; OTU: Operational taxonomic unit; PCA: Principal component analysis; PCoA: Principal coordinates analysis; PEG: Protein encoding gene; PyNAST: Python nearest alignment space termination tool; QIIME: Quantitative insights into microbial ecology; RDP: Ribosomal database project; SRB: Sulfate-reducing bacteria; VDR: Vitamin D receptor; VDRE: Vitamin D receptor element.

## Competing interests

The authors declare that they have no competing interests.

## Authors’ contributions

EC, DAA, EBC, and FM designed the experiment; EC, DAA, JMB, and LL collected and processed samples; DAA, KPK, DB, and EG analyzed the data; EC and DAA wrote the manuscript. All authors read and approved the final manuscript.

## Authors’ information

EC is a neonatologist at the University of Chicago; DAA is a microbiologist based at Argonne National Laboratory and the University of Chicago; KPK is a bioinformatics analyst based at Argonne and specializes in statistical analyses of metagenomic data; JMB is a microbiologist based at Argonne; LL is a research associate with expertise in intestinal development. DB and EG are bioinformaticians based at Argonne; EBC is a translational research investigator based at the University of Chicago; and FM is a computational biologist based at Argonne and the University of Chicago.

## Supplementary Material

Additional file 1: Table S1 Inventory of 16S rRNA-based libraries according to NEC and control groups. **Table S2.** Inventory of shotgun sequencing libraries according to NEC and control groups. **Table S3.** Shotgun read recruitment according to genome. Click here for file
